# Spring Diseases

**Published:** 1869-04

**Authors:** 


### SPRING DISEASES.

Reader! have you a mite, one solitary atom, of common sense? If you have, be persuaded to make a healthful use of it and commence on the instant. As soon as Spring begins to set in, almost everybody has more or less a feeling of lassitude; there is less buoyancy, less of an appetite, less disposition to exercise; some are so indisposed that they have to keep in the house, and numbers take to their beds. All this is your own fault; it's because you have got no sense, not a particle; or, if you have, you do not make use of it. You can readily understand that now, as the weather is warmer, you do not require as much fire in the house; and may be you are wondering why the servants will persist in making the house hotter now than in the depth of winter; they are only burning as much fuel now as in mid-winter, and they have not the sense to know this, or at least they do not care to think. The human body is a house to be kept warm; and, to be in health, its heat must be maintained at the same temperature the year round—that is, about ninety-six degrees. The stomach is, in a sense, the furnace; the food put into it is the fuel; the lungs set it on fire. Why, then, do you eat in warm weather as much as in cold weather. On a Spring day, when scarcely any fire is needed in the house, you cram as much fuel into your stomach as in the depth of winter. You see now that you have not as much sense as Biddy; she is only trying to burn up your house, you are trying to burn yourself up with fever. A baby not three months old has too much sense to poke its little finger into the candle twice, yet you are poking your whole gluttonous hulk, head foremost, every day into the furnace, and yet actually don't know what hurts you. You don't think; or, if you do, they are such diluted, milk-and-water "thinks," that a dime a load would be a bad bargain to the purchaser.

In adult life all the food we eat serves two purposes; it sustains and keeps warm. For the latter object meats, oils, butters, gravies and sweets are used; hence, in warm weather, a comparatively small amount of these things should be eaten; but in their place take breads, fruits, vegetables, melons and berries. Nature's instincts call loudly for the acids of berries and fruits, and for the earliest tender vegetables, the "greens" and the salads of our gardeners. It is because they have no heating qualities; they are rather "cooling" in their nature. They who spend much of their time indoors, would enjoy an exemption from a great many bodily discomforts if, upon the first day of Spring, they would begin to have meat for only one meal in the day, and in lessening quantities as the summer comes on. Persons who have intelligence, some skill in observing facts and making experiments, would do well to turn their attention to these things. We should aim, not merely to live, but to live in bodily health; without this, life is a burden, a failure, for an ailing man can do nothing well but—grunt and groan, and toddle about the house like a sick kitten. He is in everybody's way; he don't know what to do with himself; he can't even swallow a table-spoonful of "catnip tea" without screwing his face up into a thousand wrinkles, and then he sighs and groans as if it had half killed him.

